# The nurses’ assessment of the psychiatric care quality and the development of measures to improve it

**DOI:** 10.1192/j.eurpsy.2024.592

**Published:** 2024-08-27

**Authors:** V. Mitikhin, T. Solokhina, L. Burygina, L. Alieva

**Affiliations:** ^1^Department of Mental Health Services, Mental Health Research Centre; ^2^Psychiatric clinical hospital № 4 named after P.B. Gannushkin, Moscow, Russian Federation

## Abstract

**Introduction:**

The development of the methodology for the psychiatric care quality managing is associated with the implementation of criteria and standards, systematic evaluation and the continuous improvement of the care quality. Important role in assessing the care quality belongs to the specialists of the psychiatric services, who are both “providers” and “internal consumers” of care. At the same time, it is especially significant to take into consideration the opinion of nurses, as the largest group of specialists working in psychiatric institutions and directly providing treatment and care for patients.

**Objectives:**

To assess the quality of care by nurses of psychiatric institutions and to develop evaluation criteria and measures to improve the quality of care.

**Methods:**

Questionnaire «Assessing the satisfaction with quality of care by medical staff of psychiatric institution», including 78 questions about the quality of the structure, process and results of activities (Solokhina et al., 2014); adapted questionnaire «Assessment of the burden of psychiatric staff working in psychiatric institution», including 52 questions (WHO, 1994). The study involved 35 nurses of inpatient and outpatient services of Moscow psychiatric hospital № 4 named after P.B. Gannushkin.

**Results:**

It was found that 76,5% of respondents were satisfied with the quality of proved care in general and 78,2% of them were satisfied professional level of medical staff. The lower satisfaction was obtained when the other aspects were assessed. For example, only 58,6% of respondents were satisfied by relations with colleagues, 55,9% – by support from administration correspondingly. Dissatisfaction of nurses was related with working conditions, salary, excessive control by administration, insufficient professional training and lack of participation in the assessment of the institution’s activities.

It was revealed that the integral index of professional burden of nurses was at the average level (1,47±0,26). Inverse correlations between burden of staff and satisfaction with quality of care and institution’s activities were established. This allows to consider the professional burden as criterion in assessing the quality of care. Using obtained results, a training aimed at improving the communicative competence of medical personnel was developed and implemented in practice (Trushkina, Solokhina, 2019). For today more the 60 nurses have taken part in this training. The results demonstrate the professional growth of the participants and their communication patterns expansion.

**Conclusions:**

Nurses’ satisfaction and indicators of professional burden both can be used as criteria of assessment of the psychiatric care quality. It is also necessary to introduce in psychiatric institutions training aimed at continuous professional skills improving.

**Disclosure of Interest:**

None Declared

